# 

**Published:** 1896-02-01

**Authors:** 


Our attention has been drawn to the fact that in Dr. Wanklyn's book on "Air Analysis" the limits of permissible pollution by carbonic acid are given as six parts in 10,000, anything over eight parts being looked upon as meaning overcrowding and contamination, over ten parts being bad, and over twenty parts very bad. It seems desirable that Professor Wanklyn's published statements on the subject should have as great a publicity as the one attributed to him in regard to the recent "shelter" case, viz., that in the particular case referred to a proportion of 10·9 and 8·6 parts of carbonic acid per 10,000 of air was very good, and in no way injurious to health.

**Notices of Births, Deaths, and Marriages** are inserted in this column at a charge of 2s. 6d. for an announcement not exceeding four lines.

**Notice to Advertisers and Subscribers.**

All Advertisements, Orders for Copies of The Hospital and Business Communications should be addressed to **THE MANAGER, (Not to The Editor)**, The Hospital, 428, Strand, London, W.C.

The Cost of Subscription, post free, is as follows:—Per week, 2½d.; per quarter, 2s. 8d.; per half-year, 5s. 3d.; per annum, 10s. 6d. Foreign:—Per Annum, 15s. 2d.; payable, in all cases, in advance.

### NOTICE TO CORRESPONDENTS.

All MS., Letters, Books for Review, and other matters intended for the Editor should be addressed The Editor, The Lodge, Porchester Square, London, W.

The Editor cannot undertake to return rejected MS., even when accompanied by stamped directed envelope.

**"Burdett's Hospitals and Charities."**

Sixth year of publication. 950 pp. Scarlet cloth, gilt lettered, Price 5s. A most complete guide to British, Colonial, Indian, and American Hospitals, Dispensaries, Nursing and Convalescent Institutions, and Asylums. A few complete sets of the preceding five issues of the "Annual" may still be obtained on application to the Publishers, The Scientific Press (Limited), 428, Strand, London, W.C.

**NOTICE.—Vol. XVIII. of "The Hospital" (April to September, 1895), in handsome cloth binding, is on sale at the Office of this Journal. Price 7s. 6d. Cases for binding the Numbers contained in this Volume, 1s. 6d. Index to Vol. XVIII., 2½d., post free.**

**The Monthly Part for February is now ready, price 6d., by post, 8d.**

### SCRAPS AND GLEANINGS.

Mr. S. Gurney Buxton has sent a donation of £52 to the Norfolk and Norwich Hospital.

The annual ball at Swansea for the benefit of the hospital came off with great success.

Plans for the proposed new isolation hospital at Worcester have been accepted at an estimated cost of £7,496.

Her Majesty the Queen has forwarded ten braee of pheasants for the use of the patients of the Cancer Hospital (Free), Fulham Road, S.W.

A bazaar in aid of the West Ham Hospital was held at the Stratford Town Hall on the 15th ult. The arrangements were well carried out and the ball well attended.

Mr. Hope Morley, the treasurer of the Royal Hospital for Diseases of the Chest, City Road, E.C., has sent a donation of £50 towards the £2,000 urgently required.

An anonymous donor has given £1,000 to the Jessop Hospital for Women, at Sheffield. The late Mr. Richard Holtby, of Nafferton, has bequeathed £1,000 to the Great Driffield Cottage Hospital.

Mrs. Ernest Hart has started a new journal called *Erin*. The obje t of the paper is to advance Irish industries and excite an interest in them. *Erin* can be procured from the depôt for Irish handiwork in Wi more Street.

Through the will of the late Mr. H. B. Peart, of Brighton, the Sussex County Hospital, the Consumption Hospital at Brighton, St. Mark's Hospital for Fistula, and the Hospital for the Paralysed and Epileptic have benefited to the extent of a £1,000 legacy.

The late Mr. Reginald Ward has bequeathed £1,000 to the Royal Asylum of St. Anne's Society, which sum will be devoted to the maintenance of one child. The asylum is situated at Red-hill, where 400 children have been housed, clothed, and educated.

"The needy patients and surgical aid fund," which was alluded to in last year's report of the Budleigh Salterton Cottage Hospital as having been created by a special gift of £500 from the Rev. James Boucher, has proved a most beneficial auxiliary to the institution.

"The Guild of Kindness," established at Glasgow, is a scheme for a general assembling of Christmas toys, &c., intended for the hospitals, these to be sorted and distributed in an organised and systematic manner, after the method in which the Hospital Sunday Funds are treated.

No fewer than 10,032 patients have been treated at the Edinburgh Royal Infirmary during the year. The ordinary receipts for the twelve months under review amounted to £31,086. The managers of the infirmary are now proposing to expend about £50,000 in extending the infirmary premises.

The Emperor of Austria has issued a decree admitting women to the Medical and Philosophical Faculties of the Hungarian Universities. A clause in this new decree, however, refers to the fact that no female student will b admitted to any university without the special permission of the Minister of Justice.

The annual report of St. Mary's Cottage Hospital, Northam, near Southampton, show that there were 426 patients during the year, six only out of this number were intern patients. This hospital is entirely devoted to the treatment of persons suffering from ulcers of the leg and diseases of the skin.

The Mazawattee Ceylon Tea Company have now for many years published a charming little pocket diary. The one just issued for 1896 is even better than t e preceding ones, and contains the latest information regarding various postal, discount, and interest tables, &c. This unique diary can be obtained from all the family grocer agents of the company.

A deficiency of over £200 existed on the accounts of the Cancer Pavilion and Home, Manchester, at the end of the year, a deficiency resulting in part from expenses connected with additional furnishing, painting, and papering. During the year the hospital had provided a permanent home for 28 patients who were not able to be properly treated at home owing to the grave character of their ailments.

### THE BOOK WORLD OF MEDICINE AND SCIENCE.

SCIENCE PROGRESS. (The Scientific Press, Limited, 428, Strand, London, W.C.)

### BOOKS RECEIVED.

P. BLACKISTON, SON, AND CO., Philadelphia.
(Kegan Paul and Trench, London.)

"Manual of Gynæcology." By Henry T. Byford, M.D.

"Handbook of the Diagnosis and Treatment of Skin Diseases." By Arthur Van Harlingen, Ph.B. (Yale), M.D. Third edition, enlarged and revised.

SCIENTIFIC PRESS (LIMITED).

"Cottage Hospitals: General, Fever, and Convalescent," with many plans and illustrations. Third edition. By Henry C. Burdett.

MRS. WOSTENHOLME ELMY, Buxton House, Congleton.

"Life to Woman." By Ellis Ethelmer.

CASSELL AND CO (LIMITED).

"The Year-book of Treatment for 1896."

SWAN SONNENSCHEIN AND CO.

"Premature Burial." By Franz Hartman, M.D.

W. H. AND L. COLLINGRIDGE.

"Special Manures for Garden Crops." By Dr. A. B. Griffiths.

SANITARY PUBLISHING CO. (LIMITED).

"The Natural Arsenical Waters of La Bourboule." By A. M. Brown, M.D.

**Periodicals and Pamphlets.**—Medical Press, Scalpel, New York Medical Journal, Medical Week, Local Government Act, 1894 (Parish and District Councils), Medical Mission Quarterly, Columbus Medical Journal, Clinical Journal, Charity Organization Review, Literary Digest, Gentlewoman, Guy's Hospital Gazette, Medical Bulletin, The Therapist, Maandblad voor Ziekenverpleging, London, Girl's Own Paper, Boy's Own Paper, Sunday at Home, Leisure Hour, English Illustrated Magazine.

### THE STUDENT OF MEDICINE.

### APPOINTMENTS.

D. T. Belding, M.R.C.S., L.R.C.P.Lond., appointed Medical Officer of Health to the Dereham District Council. S. Bridger, M.R.C.S., L.R.C.P.Lond., appointed Assistant House Surgeon to the Devonshire Hospital, Buxton. T. W. Cole, B. A.Dubl., M.D., B.Ch., appointed Medical Officer of Health to the Bolsover District Council. W. Deans, M.B., C.M.Aberd., appointed Medical Officer for the Ewood Bridge District of the Haslingden Union. A. G. R. Foulerton, F.R.C.S.Eng., D.P.H.Camb., appointed Demonstrator of Biological Chemistry to the British Institute of Preventive Medicine. Dr. Fulton, appointed Medical Officer of Health to the Stevenston Parish Council. J. E. Gould, M.D.Lond., D.P.H.Camb., M.R.C.S., L.R.C.P., appointed Medical Officer of Health and Public Analyst for Bolton, and Physician to the Fever Hospital for the County Borough of Bolton. E. S. Greensill, L.R.C.P.Edin., M.R.C.S.Eng., appointed Medical Officer for the Martley Union. T. P. Greenwood, L.R.C.P.Edin., M.R.C.S., appointed Medical Officer of Health to the Easton, Kitton, &c., Rural Districts. H. C. Groves, L.R.C.P., D.R.C.S.I., appointed Medical Officer of Health for the Borough of Monmouth. C. M. Hardy, M.B., B.S.Durh., appointed Medical Officer of Health to the Croft Rural District Council. W. E. L. Horner, M.B.Lond., appointed House Physician to the Derbyshire Royal Infirmary. T. G. Kelly, B.A., M.D.Trin.Coll.Dubl., Desford, Leics., appointed Examiner to the St. John Ambulance Association. D. A. MacGregor, M.B., C.M.Edin., appointed Medical Officer of Health to the Skelmanthorpe Urban District Council. Dr. Messiter, appointed Medical Officer for the Belton District of the Thorne Union. A. Miles, M.D., F.R.C.S.Edin., appointed Surgeon to the Leith Hospital. O. Norris, L.R.C.P.I., L.M., L.S.A., appointed Medical Officer of Health for the Sherburn Rural District. R. S. Olver, M.R.C.S., appointed House Surgeon to the Derbyshire Royal Infirmary. D. Orr, M.B., C.M., appointed House Surgeon to the Edinburgh Royal Maternity and Simpson Memorial Hospital. Dr. G. L. K. Pringle, appointed Medical Officer for the Third District of the Bridgwater Union. R. J. Pye-Smith, F.R.C.S.Eng., L.R.C.P.Lond., appointed Lecturer on Surgery at the Sheffield Medical School. T. Savill, M.D.Lond., M.R.C.S., D.P.H., appointed Physician to the Hospital for Nervous Diseases, Welbeck Street. Dr. E. Shepherd, appointed Medical Officer and Public Vaccinator for the Castleton District of the Chapel-en-le-Frith Union. J. Wilkinson, M.B., C.M.Edin., appointed Medical Officer of Health to the Boston Rural District. S. Wesley Wilson, L.R.C.P.I., L.R.C.S.I., appointed Registrar Branch Medical Council (Ireland). T. R. Williams, M.B., C.M., appointed House Surgeon to the Edinburgh Royal Maternity and Simpson Memorial Hospital.

### VACANCIES.

The following vacancies are announced this week, the amount of salary in each case being given after the name of the institution:—

### Resident Medical Officers.

Chichester Infirmary.—House Surgeon. Salary £80 per annum, with board, lodging, and washing. Applications to the Secretary by February 17th.

Dundee Royal Infirmary.—Resident Clinical Assistant. Board, lodging, and washing provided. Applications to the Secretary, D. Gordon Stewart, Solicitor, Dundee, by February 5th.

Oldham Infirmary.—Junior House Surgeon; doubly qualified. Salary £50 per annum, with board and residence. Applications to E. Lionel Blake, Secretary, by February 4th.

Tower Hamlets Dispensary.—Resident Medical Officer. Salary £120 per annum, with furnished rooms, coals, gas, and attendance. Applications to the Secretary, Mr. D. F. Matheson, Tower Hamlets Dispensary, White Horse Street, Stepney, by February 1st.

### Non-Resident Medical Officers.

Metropolitan Asylums Board.—Dispenser at the Eastern Fever Hospital, The Grove, Homerton, N.E. Must not exceed 40 years of age, and qualified under the Pharmacy Act. Salary 35s. per week, with dinner daily. Applications and testimonials to the Medical Superintendent at the Hospital by February 1st.

Middlesex Hospital Medical School.—Lecturer on Anatomy and a Lecturer on Physiology. Applications to the Dean of the Medical School, Cleveland Street, W., by February 17th.

Samaritan Free Hospital for Women and Children.—Surgeon to the Out-patients. Applications to the Secretary by February 4th.

Seamen's Hospital Society, Greenwich, S.E.—Honorary Visiting Surgeon for the Branch Hospital, Royal Victoria and Albert Docks. Must be F.R.C.S.Eng. Applications to P. Michelli, Secretary, by February 3rd.

West London Hospital, Hammersmith Road, W.—Assistant Physician; must be Fellow or Member of the Royal College of Physicians of London. Applications to R. J. Gilbert, Secretary-Superintendent, by February 5th.

### UNIVERSITIES AND COLLEGES.

### University of Cambridge.

Girton College.—Alex Hill, M.D., Master of Downing College, has been appointed by the Council of the Senate a member of the governing body of Girton College.

Degrees for Women.—A memorial signed by a number of members of the Senate is in course of circulation with a view to wider support. It asks the Council of the Senate to sanction the formation of a syndicate to consider whether and under what conditions women should be admitted to the degrees of the University.

### University of Durham.

Preliminary Examination in Arts for Graduates in Medicine and Science.—The following have satisfied the examiners: J. Backhouse, W. Campbell, E. Garnsey, E. W. Jocelyne, H. Reah, F. L. Smith, B. E. Spurgin, T. Streatfield, R. L. Treble, A. Warner, H. G. Wayling, R. W. Willis.

London: The Scientific Press (Ltd.), 428, Strand, W.C.

### "THE HOSPITAL"

AN INDEPENDENT JOURNAL OF

**The Medical Sciences**

AND

**Hospital Administration,**

WITH WHICH IS ISSUED WEEKLY

A SPECIAL

### SUPPLEMENT FOR NURSES.

PUBLISHED EVERY SATURDAY.

PRICE TWOPENCE.

Matrons, Sisters, or Nurses will find in The Hospital a chronicle of the latest Nursing News, Practical Articles of the highest value to those who wish to keep themselves abreast with the times, and brightly written articles from Nurses all over the world.

**SUBSCRIPTIONS CAN COMMENCE AT ANY TIME.**

### TERMS OF SUBSCRIPTION.

### WEEKLY ISSUE.

| | Great Britain. | Foreign and Colonial. |
|---|---|---|
| For One Year ... | 10s. 6d. | 15s. 2d. |
| For Six Months ... | 5s. 3d. | 7s. 7d. |
| For Three Months... | 2s. 8d. | 3s. 10d. |

### MONTHLY PARTS.

| | Post Free to any Part of the World. |
|---|---|
| For One Year ... | 8s. 6d. |
| For Six Months ... | 4s. 3d. |
| For Three Months ... | 2s. 2d. |

Postal Orders and Cheques should be made payable to "The Scientific Press, Limited."

N.B.—In order to prevent delay, all business communications should be addressed to "The Manager" only, and not to any person individually.

### CHANGE OF ADDRESS.

In event of change of address, notice should be forwarded to the Publishers by the Saturday previous to date of publication, and the old address should also be given.

"The Hospital" can be obtained of all Booksellers and Newsagents in the United Kingdom, and at all the Railway Stalls, of Messrs. W. H. Smith and Son; and also from the following Agents in the Colonies and Abroad:—

Paris: Librairie Galignani, 224, Rue de Rivoli.
Berlin: Messrs. A. Asher and Co.
Leipsig: Mr. F. A. Brockhaus.
New York: The International News Co.
Montreal: The Montreal News Co.
Toronto: The Toronto News Co.
Melbourne, Sydney, Adelaide, and New Zealand: Messrs. Gordon and Gotch.
India: At all the Railway Bookstalls and Agencies of Messrs. A. H. Wheeler and Co.
South Africa: Messrs. Juta, Cape Town.

Publishing Offices: 428, Strand, London, W.C.

*In the Press. Price 5s.*

### BURDETT'S OFFICIAL NURSING DIRECTORY

A DIRECTORY, NOT A REGISTER.

The Hospital states: "It is well to bear in mind the difference and distinction which exists between a register and a directory. A register implies rights, and suggests that those who are not upon it are in some way or another inferior beings. But a directory is another matter. A directory gives as rights; insertion in it implies no adherence to any particular party or any particular opinion, and in no way interferes with entry on the register of any association, or the list of any institute, to which the individual may belong. All that a directory professes to do is to give information. The 'Medical Register' is not a mere list of qualified medical men; it is admission to the Register, which itself makes him qualified. For years, perhaps even now, 'Churchill's Medical Directory' contained the names of medical men who, so far as public appointments were concerned, were unqualified; it also contains the names of homœopathists, whom four out of every five medical men will not meet; and it certainly contains the names of many for whose moral character we should not like to vouch. But it gives full and accurate information about them all, and for the sake of that information it is constantly consulted both by the public and the doctors."

What Nurses Need.

"At present there is no Register for the nursing profession giving any legal status. Such a thing may come or it may not; but in either case there is no reason why there should not be a Directory, giving such information about the career of each nurse as both the public and the profession want; information, by the by, which no legal Register would be likely to insert. The 'Medical Register' is a bald and meagre thing compared with the 'Medical Directory.' What Burdett's 'Official Nursing Directory' proposes to do is to supply the want of a directory giving information. It will give no rights, it will imply no partisanship, it will give no guarantee except that what it prints is true, and is derived from official sources."

Acceptable to All Nurses.

"This ought to be enough to make it acceptable to the public, and every nurse ought to make sure that, whatever list or register she may be on, her name shall also appear on Burdett's 'Official Directory.'" Applications for forms should be made at once to the Editor, "Burdett's Official Nursing Directory," 428, Strand, W.C.

**The above work will be ready early in the Spring, and those Nurses who have not already sent in their names should at once apply for a form, which can be obtained upon application to the Publishers,**

THE SCIENTIFIC PRESS (Lim.), 428, Strand, London, W.C.

### PAMPHLETS ON NURSING.

No. 1, price 1½d. per copy, or 1s. per dozen.

**THE ADVANTAGES AND PRIVILEGES OF A TRAINED DISTRICT NURSE, AND THE DUTIES OF THE DISTRICT TOWARDS HER.**

By HENRY C. BURDETT.

No. 2, price 1½d. per copy, or 1s. per dozen.

**A GREAT MOVEMENT—THE NURSES' CO-OPERATION.**

By Mrs. FURLEY SMITH.

### NURSING BOOKS AT FULL DISCOUNT.

*LIST POST FREE.*

The Theory and Practice of Nursing (Percy Lewis, M.D.), 3/6 for **2/8.**

The Nurse's Dictionary (Honnor Morten), 2/- for **1/6.**

The Nurse's Case Book, 6d. for **3½d.**

